# Letter from Prof. John C. Dalton, Jr.

**Published:** 1852-08

**Authors:** Jno. C. Dalton

**Affiliations:** Paris


// BUFFALO MEDICAL JOURNAL

AND

MONTHLY REVIEW.

VOL. 8. AUGUST, 1852. NO. 3.

### ORIGINAL COMMUNICATIONS.

ART. I. — *Letter from Prof. John C. Dalton, Jr.*

### PHYSIOLOGICAL RESEARCHES OF M. BERNARD.

PARIS, May 26, 1852.

DEAR DOCTOR, — I consider it a great piece of good fortune to have arrived in Paris only a short time before M. Bernard commenced his course of lectures for the present year on the physiology of digestion. The high reputation of this gentleman as a scientific man, the originality and boldness of his investigations, and the novelty of many of his views, are such as to enhance very much the interest which would naturally attach to any new discovery in so important a branch of physiology, and this interest is not in the least degree abated after hearing him lecture. His appearance is that of a highly educated and intelligent man, and his style of speaking fluent, distinct, and agreeable. He excels more particularly, however, in his experiments, which are remarkably ingenious and conclusive. He does not compel his audience to take his statements on trust; but every important point bearing on his subject is demonstrated before them in such a manner that they can judge for themselves how much credit to attach to his opinions. It is this demonstrative character of his experiments, together with the ingenuity displayed in their contrivance, this combination of accurate observation with sound theory, that give so much value to his results. He himself insists particularly on the fact that experiment alone is not sufficient to determine physiological questions. It is necessary that the facts which are perceived by the senses, should be interpreted with judgment; otherwise they might often lead to false conclusions. Particularly with regard to so complicated a process as digestion, is the greatest circumspection requisite, both in contriving experiments and in drawing conclusions.

Every one is familiar with the old theories of digestion, which being almost entirely hypothetical and not based on any accurate experiments, followed each other in rotation according to the fancy of scientific men and the changes of theory; so that the process was supposed in turn to be one of an entirely vital and unexplainable character, one of simple disintegration, of coction, of fermentation and of putrefaction. The discovery of an acid fluid in the stomach, by Reaumur and Spallanzani, and more satisfactorily by Dr. Beaumont, which would dissolve alimentary substances even out of the body, replaced all these by the theory of chemical solution. A great approximation to the truth had been made, and the progress, so far as it went, was sure, since it was the result of the observation of nature. Still the theory of digestion was very imperfect. It was too *exclusive*. It attributed the process entirely to the action of an acid fluid, secreted by the walls of the stomach, which was considered as the universal solvent of all digestible alimentary substances. The peculiarity of the modern views on the physiology of digestion, and particularly of those entertained by M. Bernard, is that they regard it not as a simple but a compound process, resulting from the united action of several different secretions which are poured into the alimentary canal. These different secretions are all recognized to be digestive fluids; but digestive fluids which are destined to act successively on the food, and to digest some one portion and some another, until the whole has been reduced to a state fit for absorption into the circulation. Beside this, Bernard regards digestion not as a mere *solution* of the food by these digestive fluids, but as a process in which the food also undergoes a peculiar chemical modification. It has previously been supposed by many, that the whole object of digestion was to reduce the solid food to a fluid state in order that it might be absorbed. Liquid food, according to this idea, required no digestion. It was already capable of being absorbed, and required only to be taken into the alimentary canal to pass directly and unchanged, into the circulation. Soluble albumen, as the white of egg for example, casein in milk, or sugar in solution in water, would therefore be substances which required no digestion, but only needed to be introduced into the circulation to be employed as usual for the purposes of nutrition. Therefore these substances might be injected into the veins artificially, without ever passing through the stomach, and undergo afterward their ordinary changes in the circulation. This, however, Bernard shows not to be the case. He takes a living rabbit and empties its bladder by compressing the abdomen in the hypogastric region. By means of a syringe, furnished with a sharp pointed canula, he then injects about an ounce of a watery solution of cane sugar into the subcutaneous cellular tissue of the back, and then allows the animal to remain quiet. In a little less than an hour the bladder is again emptied, and the urine, tested by boiling with tartrate of potass and copper, shows abundant evidence of the presence of sugar; while that passed at the commencement of the experiment gave no trace of it whatever. It makes a great difference, therefore, whether sugar, in solution, be digested by passing through the stomach, or whether it be introduced directly into the circulation. In the former case it is destroyed in the blood vessels and rapidly disappears; in the latter it remains in the circulation and is excreted unchanged in the urine. It is known also by experiment that cane sugar is converted in the stomach into grape sugar, a change which appears to be necessary to prepare the sugar for the part which it is afterward to perform in the processes of alimentation. And it is a change which cannot take place in the vessels but only in the stomach; for in the above experiment of injecting cane sugar into the cellular tissue of the rabbit, it was still cane sugar which was present in the urine, and not grape sugar, as in ordinary cases of diabetes. The process of digestion, then, is one in which the food is dissolved, and at the same time undergoes a certain chemical modification; a modification which is different for different kinds of food.

Bernard's classification of the alimentary principles has nothing in it peculiar. He divides them into three classes, viz: — 1st. The albuminoid substances; containing fibrin, albumen and casein, and also the modification of albumen which exists in the gelatinous tissues, and which, after boiling, appears as gelatine. 2d. Amylaceous substance, — starch, the various kinds of sugar, and dextrine; and 3d. Fats and oils. Since all these substances exist both in animal and vegetable food, it is not necessary to suppose any essential difference in the digestive processes of carnivora and herbivora. We have animal and vegetable albumen, fibrin and casein; there is animal sugar as well as vegetable; and oily matters exist equally in both the two classes of substances. The method followed by Bernard is to investigate in detail the effect produced on the different alimentary materials by the various secretions which are poured into the digestive cavity. These fluids are five in number, viz., saliva, gastric juice, bile, and the pancreatic and intestinal fluids. They are taken up in the order in which they are met with in following the course of the intestinal tube, and in which the food is naturally subjected to their influence. He begins, then, with the saliva. In examining the properties of the saliva, Bernard is not contented with taking this fluid from the cavity of the mouth, nor even with obtaining it directly from the parotid by a salivary fistula. He collects the secretion from *all* the distinct glands, by opening the ducts and inserting a silver canula, provided with a stopper. He keeps constantly several animals (usually dogs) prepared in this way, and is enabled at any time to obtain pure saliva from the different glands, by exciting their action with sapid substances introduced into the mouth. He recognizes the existence, in this way, of four different kinds of saliva; different in their place of secretion, in their physical properties, and in the function which they are to perform, viz: 1. The saliva of the parotid; which clear, alkaline in reaction, and perfectly fluid and watery, is without the least degree of viscosity. 2. The saliva of the sublingual, which is also clear, and alkaline, but which differs from the first in being exceedingly viscous, as much so as white of egg. This saliva, when placed in a test tube will not run out by its own weight, but remains adherent to the glass. 3. The saliva of the submaxillary, which is intermediate in the degree of its viscosity between the other two. 4. The secretion of the muciparous glands of the mouth, the labial, buccal, and palatal glands, which is very viscous like the sublingual secretion, and does not differ from ordinary mucus. There might also be said to exist a fifth variety, viz., the "mixed saliva" of the mouth, resulting from the union of the four other varieties. It was on this mixed saliva that most of the earlier experiments were performed.

In following out the plan of investigation announced above, and examining the effect of saliva on the different alimentary principles by means of artificial digestions, it is ascertained that, 1st. Saliva has no chemical effect whatever on albuminoid substances; and 2d. That it is equally without influence on oily matters. There remains, then, only the class of amylaceous substances on which it can be supposed to produce any important alteration. And, in effect, it was announced some years ago, that if an emulsion of starch were treated with saliva, and the mixture kept at a moderately elevated temperature for a short time, the starch became completely converted into sugar. This experiment, which was perfectly correct so far as it went, led to the opinion that *saliva had the property of converting starch into sugar*, and that this was in reality the part it was intended to perform in the digestion of the food. This opinion, however, which is based on an experiment in artificial digestion, cannot be maintained when similar experiments are tried on the natural process. In the first place, pure saliva, taken by means of an artificial fistula from the parotid, is entirely destitute of this property. The same is true, also, of the pure saliva of the sublingual and submaxillary glands. Neither of them are capable of converting starch into sugar alone. They must all be united and the mixed saliva taken from the mucous surface of the mouth in order that the change may take place. This accounts for a discrepancy that at one time puzzled most of the experimenters on saliva, viz., that although the saliva taken from the mouth of the human subject converted starch into sugar, the saliva of the horse, taken from a parotid fistula, had no such effect. Yet it was necessary to suppose that these animals, who take so large a quantity of starch in their food, had some means of digesting it; and it was not easy to understand why the process should be different in them and in the human subject. It was simply because the pure saliva from the parotid has never any effect on starch, but only the mixed secretion, taken from the mouth.

In the second place, although saliva has this effect on starch in *artificial* digestions, it is not so in the natural process. In point of fact an emulsion of starch, taken into the stomach, is not converted by the saliva into sugar. If it were so, the starch, mixed intimately with saliva during mastication and deglutition, would rapidly disappear, and could not be detected in the contents of the stomach after a few moments. Starch mixed, out of the body, with the saliva of the mouth, and kept at the temperature of the stomach, becomes entirely converted into sugar in from seven to ten minutes. But if starch be swallowed and subjected to the natural process of digestion, it does not disappear in this rapid manner, but may be detected a long time afterward. To demonstrate this fact Bernard feeds a dog with a thick emulsion of starch which is mixed with pieces of meat, in order to render it palatable to the animal, and twenty-six minutes after the commencement of the meal, a quantity of fluid is drawn from the stomach through a fistula, which has been previously established for the purpose. This fluid, tested with tincture of iodine, shows abundant evidence of the presence of starch.

In the third place, saliva, even out of the body, cannot convert *raw* starch into sugar, but only that which has been cooked and is in the form of an emulsion. Cooked starch, in fact, is already nearly converted into sugar, and requires only a very slight influence to complete the change; so much so, that if left to itself for some days at a moderate heat, the conversion will take place without any other addition. But raw starch is neither converted into sugar spontaneously, nor can the change be effected by mixing it with saliva. Yet man is the only animal that takes starch in a cooked form. All the herbivora, that consume so large a quantity of starch in grain, potatoes, &c., take it always in a raw state. It is certain, therefore, that they must have some other means of converting this substance into sugar, than simply the influence of the salivary secretion.

Thirdly. This property of the saliva of converting cooked starch into sugar, becomes much more active in cases of mercurial stomatitis. When the saliva is in an unhealthy condition it acts more promptly than usual, and we cannot, therefore, consider that a true digestive property which is increased in activity by a morbid condition of the secretion. This property is, in fact, nothing more than an accidental one. It is considered by Bernard as owing to the change effected in the saliva by commencing putrefaction; and for this reason it is that the pure saliva from artificial fistula does not possess it, but only that which has been already sometime secreted and mixed, on the mucus surfaces of the mouth, with debris of epithelium, &c. Pure saliva, if kept for ten or fifteen days, till it shows signs of commencing putrefaction, then also begins to exhibit this property. It is a property, too, which belongs to almost all the pathological fluids of the body: of ovarian cysts, of dropsy, of peritonitis, &c., all exhibit it to a greater or less extent. The conversion of starch into sugar by the saliva, must, therefore, be regarded only as an accidental, and not as a normal physiological occurrence. During the process of digestion, as it goes on in the living body, saliva has on starch no action whatever. It appears, then, that the saliva is not the important fluid in digestion that has been supposed, having no chemical action on either albuminoid, fatty or amylaceous substances. If we suppress its secretion, in the dog or the horse, by tying the ducts of the parotid, submaxillary and sublingual glands, the *chemical phenomena of digestion suffer no change in consequence.* They go on precisely as before.

Nevertheless the saliva is certainly intended for some purpose; and, in effect, after the suppression of the secretion in the manner indicated above, it is found that the animal suffers in three particulars. A disturbance is produced in the mechanical operations of mastication and deglutition, and at the same time there is a considerable increase of thirst. It is evident that for the complete mastication and deglutition of many kinds of food it is necessary that they should be mixed with a certain quantity of fluid; and that they will require more or less fluid according to their physical conditions of dryness or moisture. It is this fluid which is supplied by the salivary glands; and it is found by experiment that during the processes of mastication and deglutition, the amount of saliva absorbed by any article of food, is in proportion to its physical characters, and has no relation with its chemical composition. The œsophagus of a horse, for example, is tied at the lower part of the neck, and opened above the ligature. A certain quantity of fluid, which has been previously weighed, is then given to the animal, and as it passes out by the opening in the œsophagus is collected and again weighed. If it was of a dry character like hay, oats, &c., it is found to have absorbed, during mastication and deglutition, from one to five times its weight of saliva. Green fodder, on the contrary, requires only half its weight; and if the food have been previously mixed with a sufficient quantity of fluid, its weight is not perceptibly increased during its passage through the mouth and œsophagus. The mode in which the saliva acts, as a whole, then, is sufficiently evident. Its importance may, also, be demonstrated by tying the salivary ducts and observing the difficulty with which certain kinds of food are afterward masticated and swallowed. A little over nine ounces of oats, e. g., were given to a horse, and the whole was masticated and swallowed at the end of ten minutes. Afterward, the ducts of the parotid glands having been tied, the same quantity of oats was again given to the animal, and it was found that the mastication and deglutition of the food was not complete at the end of three hours; so much had the difficulty of the process been increased. The quantity of saliva, however, is not only different for different kinds of food, but the different kinds of saliva are intended for somewhat different purposes. According to M. Bernard, the saliva of the parotid assists more particularly in the process of mastication; that of the submaxillary has a relation with the function of taste; and that of the sublingual, and the muciparous glands of the mouth, being more viscous and mucous in character, is required for deglutition. All these points may be demonstrated on the same animal, where we have distinct fistulæ established in the ducts of the three principal glands. On introducing into the mouth of the animal strongly sapid substances, such as vinegar or pepper, M. Bernard shows that the flow of saliva is more abundant from the submaxillary duct than from elsewhere. On the other hand, if it be a substance requiring mastication, it is the parotid which becomes excited to unusual activity; and finally, during the act of deglutition, the sublingual supplies the largest proportion.

Another proof of the speciality of function of the different glands alluded to by M. Bernard, is that they are under distinct nervous influences. If the lingual nerve be divided and its cerebral extremity irritated, the sense of taste is excited, and at the same time a flow of saliva takes place, which comes exclusively from the submaxillary.

The saliva, then, cannot be considered as taking any important part in the chemical processes of digestion, but as intended simply to assist in the purely physical operations of mastication and deglutition.

In studying the processes which take place in the digestive cavity, it is found that they become more and more chemical in their character the deeper they are situated in the interior of the body. Those which are performed in the mouth and fauces have been seen to be entirely physical in character. In the stomach, the operations are partly physical and partly chemical; — and finally, it is in the duodenum that the food suffers an alteration of a purely chemical nature.

*Gastric Juice.* — The digestive fluid which comes next in order is the gastric juice; a fluid much more important in digestion than saliva, since its action is chemical and not simply physical. The food is subjected to its action immediately on passing from the mouth, the pretended œsophagal and pharyngeal glands being no more than muciparous follicles, a continuation, as it were, of the muciparous glandulæ of the mouth, and distinct for a similar purpose.

The gastric fluid does not seem to be secreted, at least in the same proportions, by all parts of the stomach; but to be produced more particularly, if not entirely, by the pyloric portion. The secretion of the splenic portion is apparently rather of a mucous character, and destitute of the acidity and other peculiar properties which distinguish the gastric fluid proper. An ingenious experiment of M. Prevost, showed this fact directly in the dog. After establishing a gastric fistula in the animal, he evacuated the stomach, cleaned it thoroughly, by washing, and then filled it, through the fistula, with a wad of paper, or other convenient substance colored by a solution of litmus. At the end of a short time the animal was killed, and the paper was found reddened by the gastric fluid only in that portion which corresponded to the pyloric-portion. That which had filled the splenic portion of the stomach still retained entirely its blue color. It is probable that this is the case with all the simple stomachs. In animals who have a compound stomach, as the ruminantia, it is well known that the secretion of the true gastric fluid takes place only in the last; that which is nearest the pylorus. In the horse, again, we have a stomach intermediate between the simple and compound; not divided into different compartments, but still showing a striking difference in the appearance of the mucous membrane at the splenic and pyloric extremities. The splenic portion is covered with a tough and opaque epithelium; that in the neighborhood of the pylorus being softer and more mucous in character.

The existence of the gastric fluid, as has already been mentioned, was first discovered by Spallanzani and Reaumur, who obtained it by means of sponges which they compelled the animals to swallow, and which they afterward withdrew from the stomach by a cord attached to them. It is evident, however, that in this way they must have obtained an exceedingly impure fluid, and that it must have been a matter of considerable doubt how much of it came from the stomach, and how much from other parts of the alimentary canal. The question was much more completely established by Dr. Beaumont, by experiments on the person of his Canadian Voyageur, who had a gastric fistula in consequence of a gunshot wound. Dr. Beaumont took pains to extract the gastric fluid in a state of purity, by exciting the walls of the stomach, when empty, with the end of a thermometer tube, or a flexible catheter. He ascertained distinctly that when the stomach is empty of food, it contains no acid fluid whatever, but its walls are covered with a thin layer of mucus, which is neutral or alkaline in reaction; and that whenever it is excited by the presence of food, or by the irritation of a foreign body, like the gum elastic catheter, it immediately begins to secrete an acid fluid which has the property of digesting food, even out of the body. M. Bernard imitates, artificially, on animals the lesion which Dr. Beaumont was fortunate enough to encounter, accidentally, in the human subject. He takes a healthy dog, makes a short incision through the walls of the abdomen, just below, and to the left of the sternum, hooks out the anterior parietes of the stomach, and, after making in it a perforation about an inch long, inserts a short canula armed with a shoulder at each end; precisely, to use his own expression, as one would insert a stud into a shirt bosom. The canula is prevented from being drawn inward by the outer shoulder, and from being loosened from the stomach by the inner. The walls of the stomach are in this way kept in contact with the walls of the abdomen at the place of the incision. They are still further secured by one or two ligatures, the canula is provided with a cork, and the apparatus is then complete. The animal is usually entirely recovered from the effects of the wound in a few days, and is afterward ready for experiment. M. Bernard has already done the operation upon a dog in presence of his class, since the commencement of the present course of lectures, and used him afterward for the purposes of experiment.

The gastric fluid obtained in this way is clear, watery, and strongly acid. It has no marked odor, except that which is peculiar to the animal from which it was obtained. The gastric fluid of the dog, according to M. Bernard, has the following composition:

| | |
|---|---|
| Water holding an organic substance in solution, - - | 989.73 |
| Lactic and Muriatic Acids, - - - - - - | 1.95 |
| Chloride of Potassium, - - - - - - | 1.44 |
| " " Sodium, - - - - - - - | 4.59 |
| " " Calcium, - - - - - - - | 0.40 |
| " " Ammonium, - - - - - - - | 0.27 |
| Phosphate of Lime, - - - - - - - | 0.40 |
| " " Magnesia, - - - - - - - | 0.18 |
| " " Iron, - - - - - - - | 0.01 |
| | Loss, 1.03 |

Among all these ingredients of the gastric fluid there are only two which are absolutely essential. These are, first, the acid; and second, the organic matter, which is held in solution by the water. As to the acid, it is absolutely necessary that it should be present in the gastric fluid to enable it to digest food; since, if it be neutralized by the addition of carbonate of soda, this property is immediately lost. As for the particular kind of acid, it does not seem essential that it should be one or the other. If gastric fluid which has been neutralized, as above, by carbonate of soda, be again acidulated with either acetic, sulphuric, or muriatic acid, it regains completely its digestive properties. Although the gastric fluid is an acid solution, it does not act *simply* as such. For water, which is merely acidulated with muriatic acid, softens bones, by dissolving the calcareous salts and leaving behind the organic basis. Bones are affected by the gastric fluid, however, in a very different manner. The animal matter, in this case, is dissolved, and the calcareous salts left in a state of simple disintegration. A dog, fed upon bones, discharges by the bowels large quantities of nearly pure phosphate of lime; showing that this substance has not been dissolved by the gastric fluid as it would have been by a simple acidulated solution.

The gastric fluid, then, has an action which is altogether peculiar. This peculiarity is owing to the presence of the second essential ingredient, viz., the *ferment*, i. e. the organic matter, held in solution in the water, which has been known under the name of "*pepsin*." This matter, like all the ferments, is precipitable by absolute alcohol. After its precipitation, it may be redissolved in water; and if the solution be acidulated, it then exhibits the digestive property; — so that we have a kind of artificial gastric juice. In the laws which regulate its action, also, pepsin resembles the ferments. Its action is stopped, for example, by an excessively low, or excessively high temperature. At 0 (centigrade) it is inactive. At 10°× it begins to exhibit its properties, and its action becomes more and more energetic with the increased temperature, till it finally reaches its maximum 35° and 45°, above this point a further increase of heat diminishes its energy, until finally at 75°× its action is again stopped.

The active principle, then, of the gastric fluid, is an organic substance, similar in its mode of action to the ferments; and which it is necessary should be dissolved in an acidulated fluid. When these two essential ingredients, the organic principle and the acid, are both present, the gastric fluid has the property of dissolving the *albuminoid and gelatinary matters; but all other alimentary substances, amylaceous, fatty, and saccharine, are entirely unaffected by it.*

Now comes an exceedingly interesting and puzzling question. How is it that this fluid which dissolves muscular fibre, mucous membrane, cellular tissue, &c., &c., does not attack the walls of the stomach itself in which it is contained? How is it possible for a fluid, destructive of albuminoid substances, to be contained in an organ which is itself composed almost entirely of albuminoid tissues. This difficulty has usually been explained by referring to the vitality of the tissues of the stomach, by which they are rendered capable of resisting the chemical influence of the solvent; in the same manner as we know that the living body resists the extremes of heat and cold, while after death it immediately takes the temperature of the surrounding atmosphere. M. Bernard, however, explains the matter in a very different way. According to him, the gastric fluid resembles in its mode of action, not only the ferments, but also a large class of poisonous substances, such as the venom of serpents, the vaccine virus, the Woorara poison, &c., which are exceedingly active when introduced into the circulation, but still may be taken into the stomach with impunity. It is some years now since M. Bernard undertook a series of experiments for the purpose of ascertaining, if possible, the reason of this singular peculiarity; and the conclusion at which he arrived was that these poisons had no effect when taken into the stomach, simply because they *were not absorbed.* The fact had been previously explained by supposing that the gastric fluid acts upon the virus and destroys immediately its noxious properties;—that the poison is, in fact, *digested* and chemically altered immediately upon entering the stomach. Bernard, however, shows most conclusively that this is not the case. Last week he brought into the lecture-room a dog with a gastric fistula, and introducing a glass tube extracted a small quantity of gastric fluid, with which he inoculated a sparrow, by pricking it into a fresh wound in the thigh. This experiment was merely preliminary, and intended to show that the gastric fluid alone is not noxious. In effect, the sparrow appeared to suffer no inconvenience from the operation. Bernard then introduced into the stomach of the dog, about ten grains of the Woorara poison [dried extract] and allowed it to remain. Between seven and eight minutes afterward another quantity of gastric fluid was taken from the dog's stomach, and a second sparrow, of the same size and appearance as the first, inoculated with it; and at the end of two minutes the bird was dead. Notwithstanding, the dog, as well as the first sparrow, remained perfectly unharmed.

It is proved, therefore, that these poisons are not destroyed by the gastric fluid, but are simply dissolved in it and retain their activity; and that it is possible for an animal to have in its stomach, without suffering any harm, a poison which, if introduced into the circulation, would be fatal in a few seconds. Now for the reason why these substances are not absorbed and consequently do not prove injurious. M. Bernard considers the stomach as defended from their action simply by the layer of mucus which covers its internal surface; in precisely the same manner as the skin is defended by its epidermis. The Woorara cannot penetrate the epidermis, and consequently is not absorbed when held in the hand. Neither can it penetrate the mucus of the stomach and is therefore innoxious when swallowed. To exert a poisonous action it must be introduced into the circulation.

According to Bernard, the gastric fluid, also, to exert its solvent power, must be *absorbable* by the substances with which it is placed in contact; and, like the Woorara, is prevented from attacking the walls of the stomach by the mucus which covers its surface. But there is still a great difficulty remaining, viz., that as the gastric fluid is secreted by the mucous membrane of the stomach, it must necessarily be in contact with it when first produced. In order to reconcile this difficulty we must recollect the manner in which Bernard considers the gastric fluid to be formed.

There are two essential ingredients in the gastric fluid, viz., the organic matter, the ferment, called "pepsin," and the acid fluid in which it is dissolved. There are also two secretions in the stomach, viz., mucus, which is exuded from the surface of the mucous membrane, and the acid fluid which is secreted by the gastric tubules, or glandules. The layer of mucus is constantly being renewed; a fresh supply being continually exuded from the surface of the stomach, and gradually pushed farther and farther from it by that which is secreted afterward underneath. The secretion of the gastric tubules, when first produced, is simply acidulated fluid, incapable of digesting alimentary substances. It does not contain any of the organic matter.

This organic matter does not exist until the acid fluid has traversed nearly the whole thickness of the layer of mucus, and is consequently separated from contact with the mucous membrane. *The active principle of the gastric fluid is, in fact, simply the gastric mucus which has become altered while moving from the surface of the stomach toward its cavity;* which has lost, in the mean time, its viscidity, and become soluble in the acid fluid. It is, then, very easy to understand how the gastric fluid may be said to be secreted by the mucous membrane of the stomach, and yet never to have been in contact with it. So certain is it that the active digestive principle is only altered gastric mucus that we may make an artificial digestive fluid by mixing the mucus of the stomach with acidulated water. After a few days, when the mucus begins to undergo a putrefactive change, it loses its viscidity, dissolves in the water, and the solution then exhibits all the digestive properties of a true gastric fluid. It may be considered then, as almost demonstrated that the active principle of the gastric fluid and the altered mucus of the stomach are identical.

Such is the explanation of M. Bernard. It is open, perhaps, to one or two objections, though it is hardly possible to conceive of one more ingenious. It does not seem, for example, altogether clear, if the Woorara poison is prevented from entering the circulation, when swallowed, simply by the layer of gastric mucus, why opium, strychnine, belladonna, &c., &c., should not be prevented by the same means. Hydrocyanic acid, and a multitude of other poisonous substances, do not find any obstacle in this layer of mucus, when introduced into the stomach. Again, if this layer of fresh mucus is so impenetrable to the gastric fluid, it would seem almost certain to interfere very considerably with the process of digestion. When a quantity of food is taken into the stomach, and subjected to the peristaltic motion, by which it is rolled about from one extremity of the organ to the other, the mucus, which is sufficiently abundant to protect entirely the walls of the stomach, must be mixed, in no small quantity, with the food itself; and, wherever it is applied must necessarily prevent the contact of the gastric fluid, and completely interfere with the solution of the food.

The innocence of Woorara and similar poisons, when swallowed, has generally been referred to a vital property of the mucous membrane rather than to its physical condition. The mucous membrane seems to have the power of absorbing some substances in solution, and rejecting others; in the same way as the roots of plants take up only certain salts from the soil, though others, equally soluble, may be abundant in the neighborhood. It has been supposed, at the same time, as already mentioned, that it was the vitality of the stomach which defended it from the action of the gastric fluid. This idea was confirmed by the fact that immediately after death, solution of the stomach, in its own digestive fluid does, in reality, take place. The existence of all sorts of intestinal parisites, many of which are not only capable of living in the stomach of an animal, but are habitually developed there, and during a certain stage of their existence, find in the cavity of this organ the most favorable conditions of life, seemed hardly to be explained in any other way. M. Bernard, however, not only considers the impunity with which these animals inhabit the stomach as owing to another cause than their vitality, but he also denies the fact that vitality alone does protect an animal from the action of the gastric fluid. Certain substances, he says, which are perfectly digestible in themselves, are rendered indigestible by the tough and impenetrable nature of their external envelope; kidney beans, for example, in the new state, will pass through the intestinal tube undigested, because the gastric fluid cannot penetrate their coriaceous envelope. The horny epithelium of certain insects also defends them in the same manner, and enables them to live as parasites in the interior of the stomach. They are indigestible, alive and dead, for the same reason. If, however, we take an animal whose epidermis is permeable to fluids, it is no longer protected by its vitality from the action of the gastric juice. Such an animal, according to Bernard, may be digested alive. To prove this he takes a living frog, or an eel, and introducing the posterior half of the animal into a dog's stomach through the gastric fistula, fastens it in that position, with the head and anterior part of the body projecting from the opening. After an hour or more, he finds the parts which have been immersed in the dog's stomach, more or less completely digested; their vitality destroyed, and their consistency almost entirely gone. Notwithstanding, the animal is yet alive, respires, and is sensible to irritations of the skin. This experiment is a new one, brought forward by M. Bernard for the first time, at the present course. I have seen it tried several times, in the lecture room and elsewhere, but so far, I acknowledge, it has not been, in any instance, altogether satisfactory. In some it evidently failed, though the frog was left in the dog's stomach more than an hour. In all cases, too, the animal which has been subjected to this process of semi-digestion, though living at the end of the experiment, is very feeble, and dies no long time afterward. There are so many precautions to be observed, also, in so complicated an experiment, in order to avoid all sources of fallacy, that it can hardly be considered yet as entirely successful. There are, no doubt, several points in the physiology of digestion, which are still obscure. Others, however, which were formerly as much so, have been now satisfactorily settled; and a great gain has certainly been made, though something still remains to be done. There seems to be no doubt regarding the action, for example, of the first two digestive fluids. The saliva acts simply as a more or less viscous fluid, in assisting the mechanical processes of mastication and deglutition; while the gastric fluid attacks and dissolves the albuminoid substances of the food. The two remaining alimentary principles, the fatty and amylaceous, remain to be acted on by the fluids beyond the pyloric orifice.

Yours truly,

JNO. C. DALTON, Jr.

